# Rethinking vegetarianism: Differences between vegetarians and non-vegetarians in the endorsement of basic human values

**DOI:** 10.1371/journal.pone.0323202

**Published:** 2025-05-28

**Authors:** John B. Nezlek

**Affiliations:** 1 Center for Climate Change and Social Transformations, Institute of Psychology, SWPS University, Warsaw, Poland; 2 Department of Psychological Sciences, College of William & Mary, Williamsburg, Virginia, Unitd States of America; University of Palermo: Universita degli Studi di Palermo, ITALY

## Abstract

Differences between the basic human values of vegetarians and non-vegetarians were examined in three studies of samples of adults drawn from general populations, one in the US and two in Poland. Vegetarians were oversampled in the US study (514 vegetarians, 540 non-vegetarians) and in one study in Poland (301 vegetarians, 335 non-vegetarians). In the other Polish study, there 68 vegetarians and 1943 non-vegetarians. Values were measured using Schwartz’s Portrait Value Questionnaire. Across all three studies, Benevolence, Security, and Conformity values were significantly less important for vegetarians than they were for non-vegetarians, although the difference for Conformity was marginally significant (*p* < .10) in the US study. Across all three studies, vegetarians also endorsed Tradition values less strongly than non-vegetarians, although this difference was not statistically significant in the US study (*p* = .12). Across all three studies, vegetarians endorsed Stimulation, Achievement, and Power values more strongly than non-vegetarians did. There was only one value, Self-direction, for which the difference between vegetarians and non-vegetarians varied between the two countries. In the US, Self-direction values were more important for non-vegetarians than they were for vegetarians, whereas in Poland the difference was in the opposite direction. Across the three studies, there were only a few instances in which differences between vegetarians and non-vegetarians varied as a function of respondents’ gender. These results suggest that following a vegetarian diet represents a manifestation of values that emphasize independence and individuality, a possibility that is somewhat at odds with how vegetarianism is often discussed.

## Introduction

Interest in vegetarianism has increased dramatically in the past few decades [1], and this increased interest has included attention to relationships between vegetarianism and various types of values. See also Holler et al. [2] for a review. As discussed by both Salehi et al. and Holler et al., much of the research on relationships between vegetarianism and values has concerned specific values, e.g., social dominance orientation and values that underlie decisions to adopt a vegetarian diet such as ethical treatment of animals. Research on relationships between vegetarianism and values conceptualized more broadly is more limited.

Nevertheless, the specific values that have been studied can be thought of as manifestations of more basic, fundamental values that have been discussed by theorists such as Schwartz [3]. These fundamental values concern values in the broadest sense, and they are assumed to be manifested in different ways such as in attitudes and behaviors [4]. By noting this I do not intend to suggest that research on specific values is not valuable; rather, I note this to bring attention to the fact that values can be understood and analyzed at different levels of analysis. Moreover, the relative lack of research on relationships between vegetarianism and basic human values (versus research on more specific values) was an important impetus for the present study.

The study of values has a long history in psychology, and although definitions may vary, “Human values are often defined as abstract ideals that guide people’s behavior” [4]. Moreover, there are numerous models of human values that define values somewhat differently or organize values in different ways. For present purposes, values were conceptualized and measured within the context of Schwartz’s [3] model. Schwartz’s model is in widespread use and may have greater predictive validity of external criteria than other models [4].

Schwartz’s model conceptualizes basic human values in terms of 10 values, which are listed in Table 1. Although there are different measures of these values [e.g., 5,6], they all measure the same constructs/values. These 10 values are sometimes analyzed as four second-order factors, Self-transcendence, Conservation, Self-enhancement, and Openness to change, but as explained in the section Hypotheses and expectations, this was not done in the present study.

**Table 1:** Definitions of Schwartz’s basic human values.

**Benevolence**	Preservation and enhancement of the welfare of people with whom one is in frequent personal contact
**Universalism**	Understanding, appreciation, tolerance, and protection for the welfare of all people and of nature
**Conformity**	The restraint of actions, inclinations, and impulses that are likely to upset or harm others and violate social expectations or norms
**Tradition**	Respect, commitment, and acceptance of the customs and ideas that traditional culture or religion provides
**Security**	Safety, harmony, and stability of society, relationships, and self
**Stimulation**	Excitement, novelty, and challenge in life
**Self-direction**	Independent thought and action, choosing, creating, and exploring
**Achievement**	Personal success through demonstrating competence according to social standards
**Hedonism**	Pleasure and sensuous gratification for oneself
**Power**	Control or dominance over people and resources

### Basic human values and meat consumption

There is little research that has explicitly examined differences in the basic human values of vegetarians and non-vegetarians. Much of the research on relationships between values and meat consumption has examined how much meat people eat [e.g., 7], their intentions to reduce meat consumption [e.g., 8], and sometimes both [e.g., 9]. Even when vegetarianism per se is measured, it has sometimes been treated as a continuous measure, e.g., Allen et al. [10] used a 10-point bipolar scale anchored with vegan and omnivore, or vegetarians have not been distinguished from semi-vegetarians [e.g., 11, Study 1].

Although it is worthwhile to study relationships between values and meat consumption and intentions to reduce meat consumption, these are not measures of vegetarianism. As discussed by Nezlek and Forestell [12], vegetarianism is a distinct social identity. Eating less meat than average or intending to eat less meat in the future may be the first steps people take on their way to becoming vegetarians, but this does not make them vegetarians [13,14]. Along the same lines, assuming that vegetarianism and its associated identity can be represented by a continuum ignores the distinctiveness of vegetarians vs. all others (e.g., meat reducers of different kinds and omnivores).

Limiting consideration to studies that examined basic human values and that had samples of vegetarians provides a sparse basis for understanding relationships between values and vegetarianism. Dietz et al. [15] found that vegetarians were less traditional and more altruistic than non-vegetarians, and I could not find other studies that measured both constructs unambiguously. For example, Lindeman and Sirelius [11] measured Schwartz’s values and distinguished vegetarians from other dieters, but they combined vegetarians and semi-vegetarians (pescatarians) in their analyses of values (Study 1, Table 3). Regardless, they found that semi/vegetarians had higher scores than omnivores on Universalism, Stimulation, Self-direction, and Security.

Putting aside for the moment the distinctions between vegetarianism and meat reduction and between conceptualizing vegetarianism as a distinct identity vs. conceptualizing it as a continuum, the research suggests some trends. Previous research has tended to find negative relationships between Universalism and meat consumption and positive relationships between Universalism and motives to reduce meat consumption. Hayley et al. [9] and de Boer et al. [8] found that Universalism was positively related to intentions to reduce meat consumption, and similarly, Allen et al. [10] and Lehto et al. [7] found that Universalism was negatively related to meat consumption. Lehto et al. found meat consumption was positively related to Tradition and Security, and Hayley et al. found negative relationships between intentions to reduce meat consumption and Security and Power.

### Personality and vegetarianism

Although there are relationships between values and personality defined in terms of the Big Five of personality, Agreeableness, Openness, Extraversion, Conscientiousness, and Neuroticism (Emotional stability), these relationships do not tend to be large, suggesting that values and personality are distinct domains [16]. Nevertheless, research on relationships between vegetarianism and personality may be relevant to understanding relationships between vegetarianism and values.

In a recent meta-analysis of 15 studies, Reist et al. [17], concluded that vegetarians were higher on Openness and Agreeableness than omnivores. Although these traits are not the same as the values in Schwartz’s model, they are somewhat similar, at least in terms Openness. The trait of Openness shares characteristics with Schwartz’s values of Self-direction and Stimulation, which can be combined to represent a second-order value factor labeled Openness to change within the model of Schwartz and colleagues.

The trait of Agreeableness does not correspond directly to any of Schwartz’s values. Although the value of Benevolence concerns positive feelings towards others, compared to Agreeableness, Benevolence is limited to close others and a narrower scope of behaviors. Agreeableness is much more general in terms of both of these criteria. The other three personality factors, Extraversion, Conscientiousness, and Neuroticism, do not map neatly onto the values in Schwartz’s system.

### The present study

The present study was designed to examine relationships between vegetarianism and basic human values. Participants described their diets in a way that provided a basis to categorize them unambiguously as vegetarians or not, and they described their values in terms of the typology developed by Schwartz and colleagues. The analyses examined differences between vegetarians and non-vegetarians in the importance of basic human values.

### Hypotheses and expectations

Acknowledging the caveats due to the limits of the existing research, previous research suggests that vegetarians should endorse Universalism values more strongly than non-vegetarians do. Universalism includes values related to preserving the environment, and vegetarians tend to be more pro-environmental than omnivores [e.g., 18]. Universalism also includes understanding people who are non-normative, which applies to vegetarians.

I also expected that vegetarians would endorse Tradition and Conformity values less strongly than non-vegetarians. These values, along with Security, form a second-order factor that has been labeled Conservation by Schwartz and colleagues. Within most Western contexts, vegetarianism is not traditional, and so vegetarians should be less likely to endorse Traditional values than omnivores, who follow a traditional diet. Also, vegetarians do not conform to the normative omnivorous diet, so they should endorse Conformity values less strongly than omnivores. Based on the results of Lehto et al. [7], I expected that vegetarians would endorse Security values less strongly than non-vegetarians, a difference that is consistent with the expected differences in the endorsement of Tradition and Conformity values.

Extending research on relationships between vegetarianism and personality, I expected that vegetarians would endorse Self-direction and Stimulation values more strongly than non-vegetarians would. These values correspond to the personality trait of Openness, and previous research has found that vegetarians are more open than non-vegetarians. Given the lack of correspondence between Agreeableness and any of Schwartz’s values, there were no expectations based on research about Agreeableness.

As noted previously, the 10 values in Schwartz’s model can be conceptualized in terms of four second-order constructs: Self-transcendence, consisting of Universalism and Benevolence; Conservation, consisting of Tradition, Security, and Conformity; Self-enhancement, consisting of Hedonism, Power, and Achievement; and Openness to change, consisting of Stimulation and Self-direction. For three reasons, the present study examined the 10 basic human values. 1) Most previous research has conceptualized values in terms of the 10 basic values rather than the four second-order constructs. So, the analyses of the 10 values would provide results that were more comparable to previous research than the results of analyses of the four second-order constructs. 2) Given the lack of research on relationships between vegetarianism and basic human values, it would be premature to collapse across the 10 basic values and examine the second-order constructs. 3) The basic human values that compromise a second-order construct may be related to the same criteria in different ways. For example, in analyses of the 2016–2017 European Social Survey, Nezlek [19] found that Universalism was positively related to attitudes toward social minorities, the environment, and social benefits, whereas Benevolence was either negatively related or unrelated to these attitudes.

## Method

### Overview

The present paper describes the results of the analyses of three studies, one conducted in the US, referred to as US, and two studies conducted in Poland, referred to as PL-1 and PL-2. The data for each study were collected by professional survey companies. In the US and PL-1 studies vegetarians were oversampled to provide a sufficient number of vegetarians for analysis. For these two studies a question about potential respondents’ diet was used as a screener. Individuals indicated their dietary habit (which was used to classify them as vegetarians or not) at the beginning of the survey. Once the number of individuals who met a criterion (either vegetarian or non-vegetarian) had completed the survey (500 of each for the US study, 300 of each for the PL-1 study), no individuals who met this criterion were able to continue the study. Participants in all studies completed measures of human values based on the model of Schwartz and colleagues [e.g., 20].

The PL-1 study was approved by Research Ethics Committee, SWPS University, Poznan, protocol 2021-102-11. The US and PL-2 studies were approved by the Research Ethics Committee, SWPS University, Poznan, protocol 2022–149. In all three studies participants provided written (electronic) informed consent. Consent was provided by clicking on a box indicating that the individual agreed to participate. All data were deidentified before being sent to me for analysis.

The PL-1 study began on 31 August 2022 and ended on 12 September 2022. The US study began on 6 November 2022 and ended on 16 November 2022. The PL-2 study began on 16 November 2022 and ended on 28 November 2022.

### Measure of dietary habit

Participants described their dietary habit using a variant of a measure developed by Forestell et al. [21]. Participants selected one of the following options: vegan, plant-based diet, lacto-vegetarian, lacto-ovo-vegetarian, pescatarian, semi-vegetarian, occasional omnivore, or omnivore. The category plant-based diet was added to those proposed by Forestell et al. Participants were provided the following definitions:

Vegan: a person who does not eat or use any animal products (including food, clothing, cosmetics, etc.)
Plant-based diet: A person on a plant-based diet.
Lacto-vegetarian: a person who eats dairy, but does not eat eggs, fish, seafood, poultry, or red meat.
Lacto-ovo-vegetarian: a person who eats dairy and eggs, but does not eat fish, seafood, poultry, or red meat.
Pescatarian: a person who eats dairy, eggs, fish, and seafood, but does not eat poultry or red meat.
Semi-vegetarian: a person who eats fruits, vegetables, grains, dairy products, eggs, seafood, and chicken but no red meat.
Occasional omnivore: a person who occasionally eats red meat, white meat, seafood, eggs, dairy products, fruits, vegetables, and grains.
Omnivore: a person who regularly eats most meats, seafood, eggs, dairy products, fruits, vegetables, and grains.

### Participants

In all three studies, participants were classified as vegetarians if they indicated that they were vegan, a plant-based dieter, lacto-vegetarian, or lacto-ovo-vegetarian. In the PL-1 study (vegetarians oversampled) participants were limited to vegetarians, omnivores and occasional omnivores. Omnivores and occasional omnivores were considered together as non-vegetarians. In the US study (vegetarians oversampled) and in the PL-2 study (no oversampling of vegetarians) non-vegetarian participants included pescatarians, semi-vegetarians, occasional omnivores, and omnivores.

In the two Polish studies, participants indicated if they were a man, a woman, or other. In the US study response options were male, female, or other. Given that the primary analyses included sex as a between-subjects factor, only participants who selected male/female or man/woman were included in the analyses. For the US study, this meant that five participants of the original 1083 were eliminated from the analyses on this basis. For the PL-1 study and PL-2 study, no participants were eliminated on this basis.

In the US study, an additional 24 participants were eliminated from the analyses because they did not answer the dietary habit question, and another 10 participants were eliminated because they did not answer all the values questions. This left a sample of 1044, 49% vegetarians, 73% women, *M*_age_ = 50.4, *SD* = 16.1. In the PL-1 study, no participants were eliminated, and there were 636 participants, 53% vegetarians, 72% women, *M*_age_ = 39.8, *SD* = 14.5. In the PL-2 study, no participants were eliminated, and there were 2102 participants, 3.4% vegetarians, 51% women, *M*_age_ = 44.2, *SD* = 12.5. A summary of the dietary habits of participants is presented in Table 2.

**Table 2:** Dietary habits of participants for each study by sex.

Diet	US			PL-1			PL-2		
	Men	Women	Total	Men	Women	Total	Men	Women	Total
**Vegan**	91	153	244	10	56	66	7	4	11
**Plant-based diet**	28	74	102	16	57	73	7	11	18
**Lacto-vegetarian**	19	40	59	13	29	41	4	5	9
**Lacto-ovo-vegetarian**	23	86	109	20	101	121	11	19	30
**Vegetarian total**	161	353	514	59	242	301	29	39	68
**Pescatarian**	5	13	18	–	–	–	2	27	29
**Semi-vegetarian**	3	21	24	–	–	–	22	40	62
**Occasional omnivore**	28	78	106	37	79	116	230	271	501
**Omnivore**	91	301	392	81	138	219	745	697	1442
**Omnivore total**	127	413	540	118	217	335	999	1035	2034

Pescatarians and semi-vegetarians were not included in study PL-1.

### Measure of basic human values

Participants in the US study completed the PVQ-RR [6], a 57-item measure of Schwartz’s model of basic human values. Participants in both Polish samples completed the PVQ-21 [5], a 21-item measure of Schwartz’s model. The items on both versions of the PVQ ask respondents to compare themselves to a person in terms of the aspirations, goals, and so forth presented in the item.

Example items are: “Thinking up new ideas and being creative is important to her. She likes to do things her own original way,” and “Being very successful is important to him. He likes to impress other people.” As illustrated by these two examples, items are gender specific. This is done to enhance respondent’s identification with the comparison person [5]. In all studies, participants responded using the standard 6-point scale for the PVQ. The response options are 1 (*not like me at all*), 2 (*not like me*), 3 (*a little like me*), 4 (*moderately like me*), 5 (*like me*), and 6 (*very much like me*).

Consistent with established practice, prior to analysis, scores were centered on each participant’s mean response. For each participant a mean response score was calculated, and this mean was subtracted from the response for each item, a process sometimes referred to as “ipsatization.” This is done to eliminate response bias [5]. Both the PVQ-RR and the PVQ-21 were scored to provide scores on the 10 basic values originally proposed by Schwartz: Universalism, Benevolence, Conformity, Tradition, Security, Self-direction, Stimulation, Hedonism, Achievement, and Power.

The data that were analyzed in this paper are available via the Open Science Foundation repository: DOI 10.17605/OSF.IO/8ZSNE. For each study, three files are included: a fully annotated SPSS file, a CSV file, and a codebook in HTML format. This repository also contains copies of Tables S1, S2, S3, and S4.

### Overview of analyses

The data collected in each study were analyzed separately. Measures of each value were analyzed separately with a two (diet: vegetarian or not) by two (sex: male or female) ANOVA. Sex was included as a between-subjects factor to take into account the fact that more women than men are vegetarians. Without doing this, any main effects for diet could have been confounded by the unequal distributions of men and women in the two diet conditions.

### Power analysis

According to estimates generated by G*Power [22], with alpha set at .05, all three studies had power of functionally 1.0 to detect a medium effect (*f* = .25). For a small effect (*f* = .10), the US sample had power of .90, the PL-1 sample (Polish oversampling of vegetarians) had power of .71, and the PL-2 sample (Polish, no oversampling) had power of .99. These power estimates refer to the main effects for diet in the two-way ANOVA (diet by sex).

## Results

The results of the primary analyses are summarized in Table 3. When examining the means presented in Table 3, it is important to keep in mind that they are means of ipsatized scores. Positive numbers represent values that are more important than the average endorsement for a person, and negative numbers represent values that are less important than the average endorsement for a person.

**Table 3:** Results of analyses of values: Main effects for vegetarian vs non-vegetarians.

Value	Diet	US			PL-1			PL-2		
		*M*	*F*	*p*	*M*	*F*	*p*	*M*	*F*	*p*
**Universalism**	**Vegetarian**	.374	11.24	.001	.493	< 1		.461	< 1	
	**Non-vegetarian**	.262			.556			.479		
**Benevolence**	**Vegetarian**	.387	52.91	.001	.344	4.94	.002	.263	9.27	.002
	**Non-vegetarian**	.642			.549			.516		
**Conformity**	**Vegetarian**	−.193	2.95	.086	−.300	11.48	.001	−.307	6.94	.009
	**Non-vegetarian**	−.120			−.010			−.017		
**Tradition**	**Vegetarian**	−.157	2.48	.116	−.515	10.22	.001	−.431	7.11	.008
	**Non-vegetarian**	−.103			−.195			−.100		
**Security**	**Vegetarian**	.238	40.07	.001	.148	8.05	.005	.126	9.23	.002
	**Non-vegetarian**	.456			.353			.427		
**Self-direction**	**Vegetarian**	.408	17.75	.001	.403	1.02		.654	10.88	.001
	**Non-vegetarian**	.546			.332			.352		
**Stimulation**	**Vegetarian**	−.390	22.23	.001	−.247	19.89	.001	−.328	6.88	.009
	**Non-vegetarian**	−.693			−.650			−.631		
**Hedonism**	**Vegetarian**	.067	< 1		−.293	6.96	.009	−.492	< 1	
	**Non-vegetarian**	.059			−.524			−.563		
**Achievement**	**Vegetarian**	−.081	9.29	.002	−.160	12.09	.001	−.118	12.01	.001
	**Non-vegetarian**	−.225			−.459			−.505		
**Power**	**Vegetarian**	−.921	48.09	.001	−.516	9.18	.003	−.377	8.04	.005
	**Non-vegetarian**	−1.36			−.790			−.722		

The column labeled *M* contains the means for each rating. Column labeled *F* contains the F-ratio for the main effect for diet from the diet by sex ANOVA. The column labeled *p* contains the *p*-value for the corresponding *F*-ratio in the column the left. dfs were US: 1,1040; PL-1: 1,632, PL-2: 1,2098. *** *p* ≤ .001; ** *p* < .01; ^a^ *p* < .10.

This table contains only the results of the tests of the main effect of diet. Results of the tests of the main effect for sex and of the diet X sex interaction are summarized in Table S1 in the supplemental materials. Note that there were very few significant interactions of diet and sex across the three sets of analyses. There was one significant sex by diet interaction in the analyses of the US and PL-1 samples, in the analysis of Tradition in both cases, and there were no significant sex by diet interactions in the analyses of the PL-2 sample.

Across all three studies, Benevolence and Security values were significantly *less* important for vegetarians than they were for non-vegetarians. In terms of relative unimportance (negative scores), Stimulation, Achievement, and Power values were significantly *less* unimportant (i.e., more important) for vegetarians than they were for non-vegetarians. For Conformity, although the difference between vegetarians and non-vegetarians was not significant in the US study (*p* < .10), it was significant in both Polish studies, and the mean differences were all in the same direction. Similarly, for Tradition, although the difference between vegetarians and non-vegetarians was not significant in the US study (*p* = .116), it was significant in both Polish studies, and the mean differences were all in the same direction. For both Conformity and Tradition, vegetarians had lower scores than non-vegetarians.

There was only one instance in which significant differences between vegetarians and non-vegetarians were in different directions in different studies. In the US study, Self-direction values were more important for non-vegetarians than they were for vegetarians, whereas in both Polish studies the opposite occurred: Self-direction values were more important for vegetarians than they were for non-vegetarians, although this difference was significant in only the PL-1 study.

Universalism combines three values: tolerance, concern for others, and preservation of the natural environment. Given that concerns about the environment are important reasons why many people adopt a vegetarian diet [e.g., 23], the environmental values component of Universalism was analyzed separately. For the PVQ-RR (US study) this was a three-item scale, and it was a single item for the PVQ-21 (the two Polish studies). As before, these analyses were two (diet) by two (gender) ANOVAs. The results of these analyses were similar to the results of the analyses of Universalism: a significant effect for diet in the US study (*p* < .001) and non-significant effects for diet in the two Polish studies (both *p*s > .15). Environmental values were more important for vegetarians than they were for non-vegetarians.

Note: All significant effects remained significant after controlling for multiple comparisons using the Benjamini-Hochberg correction [24].

### Exploratory analyses: Comparisons among vegetarians, among non-vegetarians, and people who do not eat red meat

The present studies were designed to compare the basic human values of vegetarians and non-vegetarians. Nevertheless, the data that were collected provided a basis to examine differences among vegetarians and differences among non-vegetarians. One set of analyses compared vegans to non-vegan vegetarians and another compared omnivores and occasional omnivores. A third set of analyses compared people who do not eat red meat with vegetarians and with omnivores.

These analyses were done to contribute to the existing database of research on individual differences related to dietary habit. Given their post-hoc nature, there were no hypotheses regarding the differences between the groups examined in these analyses. Moreover, the results of these analyses need to be evaluated within the context that the study was not designed to examine such differences. All medium effects were defined as they were previously, *f* = .25.

#### Vegans vs. vegetarians.

Although vegans are vegetarians, they are distinct from non-vegan vegetarians in that vegans do not consume or use any animal-based products [25]. In contrast, although non-vegan vegetarians may not eat flesh, they may consume or use animal-based products such as eating eggs or wearing leather shoes and wool sweaters. To determine if the basic human values of vegans and non-vegan vegetarians differed, the data from the US and PL-1 studies were analyzed. In both of these studies, vegetarians were oversampled, which provided adequate statistical power for tests comparing vegans and non-vegan vegetarians (power for a medium effect: US study, .99; PL-1 study, .94). In the PL-2 study, there were only 11 vegans which did not provide a basis for comparing differences between vegans and non-vegan vegetarians.

The results of these analyses, two-way ANOVAs diet (vegans or non-vegan vegetarians) by sex (male or female) were inconsistent across the two samples. In the PL-1 study, there were no significant differences between vegans and non-vegan vegetarians. In the US study, vegans and non-vegan vegetarians differed in how strongly they endorsed Universalism, Benevolence, Conformity, Self-direction, and Power values. Non-vegan vegetarians endorsed Universalism. Benevolence, and Self-direction values more strongly than vegans did. Conformity and Power values were more *unimportant* for non-vegan vegetarians than they were or vegans. The results of these analyses are summarized in Table S2 in the supplemental materials.

#### Omnivores vs. occasional omnivores.

Differences in how strongly omnivores and occasional omnivores endorsed basic human values were examined in all three samples. These analyses were two (diet: occasional omnivore or omnivore) by two (sex: female or male) ANOVAs. For the US study, these analyses included 495 participants (power for a medium effect, .99). 235 participants in the analyses of the PL-1 study (power for a medium effect, .97), and 1943 participants in the analyses of the PL-2 study (power for a medium effect, functionally 1.0). Note power is estimated for the main effect for the two-way ANOVAs.

The results of these analyses are summarized in Table S3, which is in the supplemental materials. The only difference that was found consistently in these analyses was that occasional omnivores endorsed Universalism values more strongly than omnivores in all three studies. Other than these differences, there were only two other significant differences across all three studies, and these were found for different values. In the PL-2 study Tradition was more *unimportant* for occasional omnivores than it was for omnivores, and in the PL-1 study, and Hedonism was more *unimportant* for occasional omnivores than it was for omnivores.

#### Vegetarians, omnivores, and people who do not eat red meat.

For these analyses, vegetarians were defined as vegans, plant-based dieters, lacto-vegetarians, and lacto-ovo-vegetarians. Pescatarians and semi-vegetarians were classified as eat no red meat, and omnivores were defined as occasional omnivores and omnivores. The analyses were three (diet) by two (sex) ANOVAs with planned contrasts of vegetarians vs. eat no red meat, and eat no red meat vs. omnivores. The data collected in the PL-1 study were not analyzed using this model because pescatarians and semi-vegetarians were not included in the PL-1 study. The results of these analyses are summarized in Table S4, which is in the supplemental materials.

This summary contains a column labelled “Overall” which contains the results of the significance tests of the main effect of diet. These main effects are similar to the main effects for diet reported in the original analyses (vegetarians vs non-vegetarians) primarily because compared to the number of omnivores there were relatively few pescatarians and semi-vegetarians. This meant that the means for non-vegetarians in the original analyses were not influenced that strongly by the responses of pescatarians and semi-vegetarians.

The results of the contrast analyses did not provide a basis for drawing a conclusion about the extent to which those who do not eat red meat were more similar to vegetarians or to omnivores or to neither. There was no value for which the means for participants who did not eat red meat differed significantly from the means for omnivores in both the US and PL-2 studies. There was only value (Power) for which the difference between vegetarian and those who do not eat red meat were significant in both studies. Power was more unimportant for those who do not eat red meat than it was for vegetarians.

## Discussion

Across all three studies, Benevolence, Security, and Conformity values were significantly less important for vegetarians than they were for non-vegetarians, although the difference for Conformity was marginally significant (*p* < .10) in the US study. Across all three studies, Stimulation, Achievement, and Power values were significantly more important for vegetarians than they were for non-vegetarians. These differences suggest that people who adopt a vegetarian diet are independent thinkers who are not afraid to “march to the beat of a different drum.”

As discussed previously, vegetarians are a minority in many countries, including the countries in which the present studies were conducted, and their identification as member of this minority is an act of choice. People choose to eat meat or not. This means that vegetarians can be seen by members of the omnivorous majority as individuals who have explicitly rejected the majority diet and the values for which it stands. In extremis, such perceptions can be manifested in what has come to be called “vegetarian threat,” a constellation of beliefs held by omnivores that vegetarians pose a threat to society and its way of life [26].

In the face of such beliefs and in the face of the experiences of rejection and criticism that may accompany the expression of such beliefs [27,28], vegetarians need to be committed and hold their beliefs strongly. This strength is reflected in the fact that vegetarians view their diets as a more important part of their sense of self than omnivores do [29,30]. The present results suggest that adopting a vegetarian diet is characterized by a strong commitment and a relative de-emphasis on conforming to existing norms, i.e., following an omnivorous diet.

Nevertheless, the present results are somewhat inconsistent with the conclusion one might draw from research on gender-roles and vegetarianism. More women than men are vegetarians [e.g., 31], and the consumption of meat is associated with masculinity [32]. Research on gender-role stereotypes suggests that women are more interpersonally-oriented than men are, whereas men are more task-focused than women are, and this research also suggests that women are more prosocial than men are [33]. Considering research on gender/vegetarianism and research on gender roles together suggests that compared to non-vegetarians, vegetarians should be more benevolent, more concerned about security, more concerned about conformity (harmony, “fitting in,” etc.), and less concerned about achievement and power. This was clearly not the case in the present study.

Although at first glance, the lack of correspondence between the present results and research on perceptions of vegetarians as less masculine may seem problematic, it is not. Research on perceptions of vegetarians concerns how vegetarians are seen, not how they are. The fact meat-eaters may see vegetarianism as “feminized” (within the context of gender-role stereotypes) simply and only concerns perceptions of vegetarians. As it turns out, the present results suggest that vegetarians may not only not be feminized (in terms of traditional sex role stereotypes), but in terms of the values of Schwartz’s model interpreted in terms of gender-role stereotypes, vegetarians may be more masculinized than meat-eaters (endorsing power values, achievement values, seeking stimulation, etc.).

### Comparing the present results with the results of past research

As discussed in the introduction, comparing the present results with the results of previous research is not straightforward because few studies have examined differences in basic human values between vegetarians and non-vegetarians (considered as groups). One study that did examine differences between vegetarians and non-vegetarians in terms of values, Dietz et al. [15], found that vegetarians were lower on endorsing Tradition values than non-vegetarians, a finding similar to the present results.

Despite differences in exactly what was studied, the present results comport with previous research in some ways. For example, similar to the present results, Lehto et al. [7] found that endorsing Tradition and Security values were positively related to meat consumption. Consistent with the difference between vegetarians and non-vegetarians found in the present study, Hayley et al. [9] found a negative relationship between endorsing Security values and intentions to reduce meat consumption.

On the other hand, the present results do not comport with the results of previous research in some ways. For example, Hayley et al. [9] found that endorsing Power values was negatively related to intentions to reduce meat consumption. In contrast, in the present studies, vegetarians had higher scores on Power values than non-vegetarians, a difference that represents the opposite relationship from that found by Hayley et al.

Consistent with previous research, vegetarians were higher on Universalism than non-vegetarians in the US study. In contrast, in both Polish studies, this difference was not significant, a null result that could not be attributed to a lack of power, at least in one of the Polish studies assuming a small effect. The differences between vegetarians and non-vegetarians in Self-direction also varied between the two countries. In the US study, Self-direction was *less* important for vegetarians than it was for non-vegetarians, whereas in the two Polish studies Self-direction was *more* important for vegetarians than it was for non-vegetarians.

The reason for these difference is not clear. The US can (broadly) be described as an Anglo-Saxon culture, whereas Poland can be described as Western Slavic. The two countries also have different modern histories in terms of political systems. Nevertheless, it is not immediately obvious how such differences would be manifested in differences between vegetarians and non-vegetarians in terms of the importance of Self-direction values and Universalism values. Determining the extent to which differences between the values of vegetarians and non-vegetarians vary as a function of culture will require research specifically designed to do this.

### Comparisons based on type of meat restriction

On an exploratory basis I examined differences in the values of participants as a function of the type of meat restriction they practiced. Compared to the differences that were found between vegetarians and non-vegetarians defined in terms of whether people ate animal flesh or not, these analyses did not find consistent differences. Although the values of vegans were different from the values of non-vegan vegetarians in the US sample, they were not different in the Polish sample. There were few differences between omnivores and occasional omnivores (people who may have been trying to reduce their consumption of red meat). Analyses that compared those who did not eat red meat with vegetarians and with omnivores did not find a clear pattern of differences.

The lack of clear patterns in the results of these additional analyses suggests that a distinction between vegetarians and non-vegetarians based on the consumption of flesh of any kind is the most important in terms of understanding people’s values. Although vegans and non-vegan vegetarians may differ in some ways, the fact that they have decided not to eat animal flesh may be a tie that binds them in terms of common values. In parallel, although pescatarians, semi-vegetarians, occasional omnivores, and omnivores may differ in some ways, they all eat flesh of some kind, and this may be a tie that binds them.

Nevertheless, conclusions about such differences are preliminary/tentative because the study was not designed to examine such differences. For example, the sample sizes for some dietary habits were too small to provide a firm basis to draw conclusions. Given the increasing attention that meat reduction is receiving among researchers and the public at large, it would seem that understanding the values underlying meat reduction is important.

### Conclusions, limitations, and future directions

The present study is the first of which I am aware that compares the basic human values of vegetarians and non-vegetarians in different cultural groups. Although the present study breaks new ground in doing so, it is not certain how the present results might generalize to other cultural groups, e.g., South or Latin America or Asia. This is an empirical question. There is also the issue of how values were defined/measured. Although Schwartz’s typology is in widespread use, measuring values within other conceptual models might lead to different conclusions about differences in the values of vegetarians and non-vegetarians.

Most important perhaps are questions about how/if basic human values lead to the adoption of vegetarian diets. Why does holding Traditional and Benevolence values reduce the likelihood someone will be a vegetarian? Why does endorsing Power values make it more likely someone will be a vegetarian? Although Schwartz’s model suggests that values are causes of behaviors such as dietary habit, is this necessarily the case? Perhaps people adopt vegetarian diets and then their values change.

There is also the issue of people who are not vegetarians per se, but who do not eat red meat or do not eat meat on a regular basis, either by plan or circumstance. The results of the present analyses did not provide a basis for thinking of these people as more like vegetarians or omnivores, nor as omnivores in transition to vegetarianism. The present study was not designed to examine these groups per se, and similar to any null results, the present null results need to be viewed cautiously.

Regardless, the present results suggest that although vegetarians may be more sensitive to the pain and suffering of animals and may be more aware of threats to the environment than non-vegetarians, this sensitivity and awareness do not reflect basic human value of benevolence, at least as defined by Schwartz and colleagues. Moreover, the present results suggest that vegetarians hold values consistent with being members of a social minority who are willing to stand by their principles. Although the present studies leave important questions unanswered, they suggests a path forward.

## Supporting information

S1 Table Summary of sex effects. (PDF)

S2 Table Values of non-vegan vegetarians and vegans. (PDF)

S3 Table Values of occasional omnivores and omnivores. (PDF)

S4 Table Vegetarians, red-meat avoiders, and omnivores. (PDF)
