# Supplementary material for: Rethinking vegetarianism: Differences between vegetarians and non-vegetarians in the endorsement of basic human values
Source: PLoS One. 2025 May 28;20(5):e0323202. doi: 10.1371/journal.pone.0323202 (PMC12118818; doi:10.1371/journal.pone.0323202)
Supplement: S1 Table — (PDF) [file pone.0323202.s001.pdf]

Table S1. Summary of sex effects

	US					PL1					PL1			
	**Men**	**Women**				**Men**	**Women**				**Men**	**Women**		
**Sample size**	285	759				177	459				1028	1074		
	**Means**		**p-values**			**Means**		**p-values**			**Means**		**p-values**	
**Measures**			**Sex**	**Diet-Sex**				**Sex**	**Diet-Sex**				**Sex**	**Diet-Sex**
**Universalism**	.296	.340	.192	.238		.441	.608	.013	.099		.341	.599	.001	.312
**Benevolence**	.455	.574	.001	.491		.393	.500	.106	.066		.242	.537	.000	.087
**Conformity**	-.119	-.194	.079	.505		-.073	-.236	.057	.341		-.168	-.157	.920	.476
**Tradition**	-.101	-.159	.086	.826		-.203	-.507	.002	.046		-.194	-.337	.248	.504
**Security**	.278	.416	.000	.011		.230	.272	.561	.826		.201	.352	.127	.569
**Self-direction**	.519	.435	.011	.033		.291	.444	.031	.118		.523	.483	.663	.583
**Stimulation**	-.501	-.582	.205	.940		-.433	-.464	.739	.228		-.348	-.611	.023	.142
**Hedonism**	.057	.068	.818	.342		-.395	-.421	.767	.084		-.456	-.598	.228	.238
**Achievement**	-.186	-.120	.164	.152		-.277	-.343	.440	.425		-.216	-.406	.090	.371
**Power**	-1.079	-1.205	.048	.389		-.605	-.701	.291	.424		-.407	-.692	.019	.533

Note: Columns labeled “Sex” contain the *p*-value for the main effect for sex. Columns labeled “Diet-Sex” contain the *p*-value for the diet by sex interaction.
