# Supplementary material for: Rethinking vegetarianism: Differences between vegetarians and non-vegetarians in the endorsement of basic human values
Source: PLoS One. 2025 May 28;20(5):e0323202. doi: 10.1371/journal.pone.0323202 (PMC12118818; doi:10.1371/journal.pone.0323202)
Supplement: S2 Table — (PDF) [file pone.0323202.s002.pdf]

Table S2. Endorsement of values by non-vegan vegetarians and vegans

		US		PL-1	
		M	***F*-ratio**	***M***	***F*-ratio**
**Universalism**	**Non-vegan**	.451	11.02**	.464	< 1
	**Vegan**	.300		.596	
**Benevolence**	**Non-vegan**	.488	20.54***	.321	< 1
	**Vegan**	.270		.454	
**Conformity**	**Non-vegan**	-.280	7.96**	-.269	1.15
	**Vegan**	-.112		-.461	
**Tradition**	**Non-vegan**	-.172	< 1	-.564	1.26
	**Vegan**	-.149		-.325	
**Security**	**Non-vegan**	.246	< 1	.160	< 1
	**Vegan**	.222		.058	
**Self-direction**	**Non-vegan**	.489	12.57***	.399	< 1
	**Vegan**	.324		.438	
**Stimulation**	**Non-vegan**	-.431	1.59	-.261	< 1
	**Vegan**	-.322		-.180	
**Hedonism**	**Non-vegan**	.042	< 1	-.263	< 1
	**Vegan**	.098		-.399	
**Achievement**	**Non-vegan**	-.084	< 1	-.135	< 1
	**Vegan**	-.077		-.245	
**Power**	**Non-vegan**	-1.085	14.56***	-.458	2.35
	**Vegan**	-.729		-.750	

Note: *** $p \leq .001$; ** $p < .01$. For the US sample, there were 266 non-vegan vegetarians and 244 vegans; for the PL-1 sample, there were 235 non-vegan vegetarians and 66 vegans.
