# Supplementary material for: Rethinking vegetarianism: Differences between vegetarians and non-vegetarians in the endorsement of basic human values
Source: PLoS One. 2025 May 28;20(5):e0323202. doi: 10.1371/journal.pone.0323202 (PMC12118818; doi:10.1371/journal.pone.0323202)
Supplement: S3 Table — (PDF) [file pone.0323202.s003.pdf]

Table S3. Endorsement of values by occasional omnivores and omnivores

		US		PL-1		PL-2	
		M	*F*-ratio	*M*	*F*-ratio	*M*	*F*-ratio
**Universalism**	**Occasional omnivore**	.357	4.56*	.729	9.59**	.531	6.69**
	**Omnivore**	.228		.474		.446	
**Benevolence**	**Occasional omnivore**	.626	< 1	.569	< 1	.501	< 1
	**Omnivore**	.660		.538		.521	
**Conformity**	**Occasional omnivore**	-.061	1.58	-.044	< 1	-.023	< 1
	**Omnivore**	-.155		.008		.003	
**Tradition**	**Occasional omnivore**	-.087	< 1	-.252	< 1	-.181	7.55**
	**Omnivore**	-.101		-.164		-.040	
**Security**	**Occasional omnivore**	.452	< 1	.435	1.62	.449	< 1
	**Omnivore**	.455		.312		.433	
**Self-direction**	**Occasional omnivore**	.495	1.15	.380	1.44	.344	< 1
	**Omnivore**	.556		.303		.334	
**Stimulation**	**Occasional omnivore**	-.708	< 1	-.708	< 1	-.590	1.82
	**Omnivore**	-.699		-.620		-.654	
**Hedonism**	**Occasional omnivore**	.057	< 1	-.747	8.86**	-.612	1.62
	**Omnivore**	.056		-.413		-.549	
**Achievement**	**Occasional omnivore**	-.312	1.68	-.520	< 1	-.448	2.90[a]
	**Omnivore**	-.196		-.431		-.527	
**Power**	**Occasional omnivore**	-1.384	< 1	-.797	< 1	-.731	< 1
	**Omnivore**	-1.361		-.793		-.717	

Note: *** $p \leq .001$; ** $p < .01$; [a] $p < .10$. For the US sample, there were 105 Occasional omnivores and 390 Omnivores; for the PL-1 sample, there were 116 Occasional omnivores and 219 Omnivores; for the PL-2 sample, there were 501 Occasional omnivores and 1442 Omnivores.
