# Supplementary material for: Rethinking vegetarianism: Differences between vegetarians and non-vegetarians in the endorsement of basic human values
Source: PLoS One. 2025 May 28;20(5):e0323202. doi: 10.1371/journal.pone.0323202 (PMC12118818; doi:10.1371/journal.pone.0323202)
Supplement: S4 Table — (PDF) [file pone.0323202.s004.pdf]

Table S4. Endorsement of values by vegetarians, people who do not eat red meat, and omnivores

		Vegetarian	No Red Meat	Omnivore	Overall	Veg v. NoRed	NoRed v. Omni
**Universalism**	**US**	.374	.319	.256	6.04**	< 1	< 1
	**PL-2**	.461	.674	.468	3.51*	3.75*	7.00**
**Benevolence**	**US**	.387	.484	.653	27.67***	< 1	2.69a
	**PL-2**	.263	.436	.516	5.01**	2.31	< 1
**Conformity**	**US**	-.193	.104	-.135	3.38*	5.81*	3.71*
	**PL-2**	-.307	-.197	-.007	5.13**	< 1	3.14a
**Tradition**	**US**	-.157	-.160	-.098	1.49	< 1	< 1
	**PL-2**	-.431	-.467	-.078	8.97**	< 1	10.43**
**Security**	**US**	.238	.480	.455	20.28***	5.86*	< 1
	**PL-2**	.126	.264	.437	6.37**	1.04	3.24a
**Self-direction**	**US**	.408	.599	.542	9.01***	4.00*	< 1
	**PL-2**	.654	.594	.338	9.87***	< 1	8.35**
**Stimulation**	**US**	-.390	-.581	-.703	11.44***	1.05	< 1
	**PL-2**	-.328	-.432	-.638	5.11**	< 1	3.36a
**Hedonism**	**US**	.067	.092	.057	< 1	< 1	< 1
	**PL-2**	-.492	-.502	-.564	< 1	< 1	< 1
**Achievement**	**US**	-.081	-.278	-.222	2.18**	2.07	< 1
	**PL-2**	-.118	-.479	-.505	6.02**	5.57*	< 1
**Power**	**US**	-.921	-1.313	-1.365	23.87***	4.48*	< 1
	**PL-2**	-.377	-.749	-.721	4.05*	4.99*	< 1

Note: *** $p \leq .001$; ** $p \leq .01$; * $p \leq .05$; [a] $p < .10$. In the US sample, there were 510 vegetarians, 41 no red meat eaters, and 493 omnivores. In the PL-2 sample, there were 68 vegetarians, 91 no red meat eaters, and 1943 omnivores.
